# Comparative analysis of immune related genes between domestic pig and germ-free minipig

**DOI:** 10.1186/s42826-020-00077-7

**Published:** 2020-12-01

**Authors:** Ju Young Lee, Sang Eun Kim, Hoon Taek Lee, Jeong Ho Hwang

**Affiliations:** 1 Animal Model Research Group, Korea Institute of Toxicology, 30, Baekhak 1-gil, Jeongeup-si, Jeollabuk-do 143-701 South Korea; 2 Division of Human and Environmental Toxicology, University of Science & Technology, Daejeon, South Korea; 3 Department of Stem Cell and Regenerative Biotechnology, Konkuk University, 120 Neungdong-ro, Gwangjin-gu, Seoul, 05029 Republic of Korea

**Keywords:** Minipig, Germ-free facility, Differentially expressed gene, Systemic immune activation

## Abstract

Recently, minipig has been considered as an animal model that is appropriate for human disease model to study toxicology, pharmacology, and xenotransplantation. Nevertheless, minipigs are bred in various environment according to their use. Here, we suggest that minipigs used for research should be bred in well-controlled facility, comparing immune status of pigs raised in different breeding environment. DNA microarray was performed with ear skin and placenta of Landrace domestic pigs (DPs) and Minnesota germ-free minipigs (GPs). Their immune transcriptome was analyzed by gene ontology (GO) annotation database, based on criteria of |log_2_ fold change| ≥1 with *P* ≤ 0.05. As a result, we found that immune related genes in the ear skin of DPs were highly activated, compared to GPs. On the other hand, no significant s were found in the placenta. Quantitative real-time PCR (qRT-PCR) was performed in five candidate immune genes. Their fold changes were consistent with the results from DNA microarray (*P* ≤ 0.05). In conclusion, we experimentally proved that porcine immune system was affected by different breeding environment, suggesting the importance of controlling microbes in animal room for the qualified research.

**Supplementary Information:**

The online version contains supplementary material available at 10.1186/s42826-020-00077-7.

## Introduction

Experimental animal models have been considered as important research tool to conduct preclinical study and identify human disease mechanism. The species of animal model have been also diversified, such as rodents, non-rodents, and non-human primates [1]. In general toxicity test, one species of non-rodents is essentially required to meet Good Laboratory Practice (GLP) guideline [2]. However, arguments to utilize those animals for research have been constantly raised due to ethical issues and lacks of experiment reproducibility [3]. To overcome these problems, the minipig has been raised as an alternative animal due to similarity of pathological features with human, and being relatively free from animal ethic issues. The advantages of utilizing minipig are as follows [4]. First, the minipig is relatively free from animal ethic issue than other animals because of their industrial use as the livestock in human society. Second, the minipig has been improved for proper size to be managed and bred easier than traditional farm pigs, which enables to reduce economic costs incurred by securing breeding space for installment of cages and their feed consumption. In addition, these features produce reliable data for researcher by increasing the number of animals in experiment. Finally, the minipig shares similar histological and physiological characteristics with human, which makes them as standard resource for xenotransplantation. Despite these advantages, it is highly necessary to breed minipigs in the well-controlled facility such as Specific Pathogen Free or Germ-free grade [5]. Immunogen, such as bacteria, in the conventional facility could bring out infection and induce activation of immune response, which leads to affect experiment data. Moreover, a recent report warns of transmission of zoonotic disease from porcine organ to human patient through xenotransplantation [6].

Thus, it should be experimentally identified that immunological change of pigs in different breeding environment for researchers to perform safe and qualified experiments. We chose ear skin, primary immunological barrier against external environment, as target tissue [7]. Placenta known for immune privilege region was chosen as control tissue to confirm endogenous variation caused by immunological feature of tissues [8]. Transcriptome profiling and comparative analysis between domestic pigs (DPs) and germ-free minipigs (GPs) were performed using DNA microarray and quantitative real-time PCR (qRT-PCR).

## Methods/experimental

### Animals

**Fig. 1:**
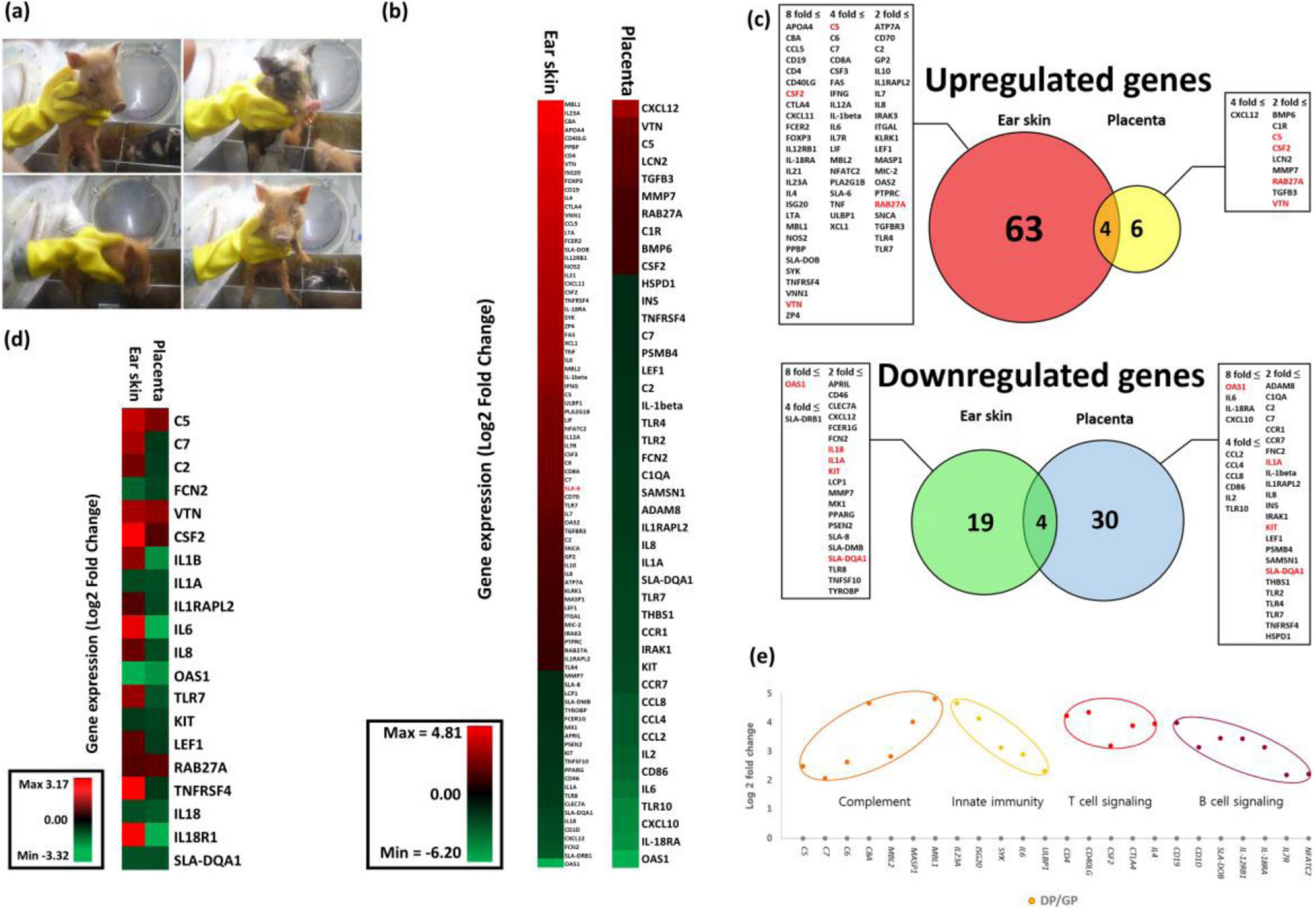
Immunological transcriptome profile of pigs. **a** Pictures of Germ-free piglets born in sterile isolator. **b** Heatmap for comparison of immune related genes in ear skin and placenta. Each fold change was calculated by comparing domestic pigs (DPs) with germ-free minipigs (GPs). **c** Venn Diagram showing up and down-regulation of immune-related genes in DPs compared to GPs. The number of genes and gene symbols are described in each circle and box. Genes having same expression pattern in both tissues are marked as red color. **d** Heatmap for comparing pattern of immune-related genes commonly expressed in both tissues. **e** Process of systemic immune activation of DPs. The same colored dots are grouped by same signaling pathway. The Y-axis indicates the log2 fold change of relative gene expression in DPs compared to GPs. The X-axis indicates gene symbol

A pregnant Minnesota minipig raised in sterile condition (Laboratory Animal Research Center in Konkuk University, Seoul, Korea) was randomly selected from pregnant sows. The facility continuously maintained positive pressure with HEPA-filtered air, and temperature (22 ± 2 °C) and humidity (50 ± 5%). Newborn germ-free piglets (3 males and 1 females; *n* = 4) were produced by hysterectomy of pregnant sow under aseptic conditions. Average body weights of the piglets ranged from 450 to 550 g. They were transferred to the aseptic isolator (1200 W × 900D × 950H m/m, SK-ISO1700HBP600; Three-shine Inc., Daejeon, Korea), and fed sterilized soy bean milk (Fig. 1a). The procedures that contained animal welfare and ethical problem were approved by the Konkuk University Institutional Animal Care and Use Committee (IACUC; approval number: KU01410). A pregnant Landrace pig raised in conventional condition (Dongsan Farm, Younchun, Gyunggido, Korea) was also selected from pregnant sows. The average body weights of domestic piglets (3 males and 1 females; *n* = 4) ranged from 1.05 to 1.25 kg. All breeding process for the piglets were performed under same condition with that for Germ-free piglets except controlling microbial environment.

### RNA sample preparation

Chorion parts of placenta (*n* = 4) were specifically collected and frozen with liquid nitrogen immediately at birth. Ear skins of germ-free piglets (*n* = 4) were obtained in the aseptic isolator on 3 weeks after birth. To prevent RNA degradation, frozen placenta samples are emerged in 1 ml of a RNA*later*-ICE (Ambion, Invitrogen Carlsbad, CA, USA) solution and incubated in 4 °C for over-night. Total RNA was extracted from the ear skins and the placentas by using Phenol/Chloroform extraction method. All RNA samples were pooled for DNA microarray. Reverse transcription reaction for qRT-PCR was performed with a QuantiNova Reverse Transcription Kit (QIAGEN Science Inc., Germantown, MD, USA).

### DNA microarray and gene ontology analysis

DNA microarray was performed using Porcine (v2) Gene expression 4 × 44 K microarray (Cat.no G2519F-025440; Agilent technology, Santa Clara, CA, USA) covered with 43,803 probes in capable of detecting every porcine gene. The procedure was followed by technical manual of SurePrint Gene Expression Array (Agilent technology). Quality check of RNA samples was performed using Agilent 2100 Bioanlayzer (Agilent technology). Gene Ontology (GO) was analyzed by using DAVID Bioinformatics Resources 6.87 [9].

### Quantitative real-time PCR

qRT-PCR was performed in 20 μl PCR reaction mixtures that included 2x iQ SYBR Green Supermix (Bio-rad Laboratories. Hercules, CA, USA, each primer at a concentration of 0.5 μM, and template cDNA according to the manufacturer’s protocol. it was run in a QuantStudio 5 Real-Time PCR System (Applied Biosystmes, Foster city, CA, USA) using the amplification parameters: the initial denaturation step at 95 °C for 3 min was followed by 40 cycles of denaturation at 95 °C for 20 s, annealing and elongation at 60 °C for 1 min without the final elongation step. Gene expression level of Glyceraldehyde 3-phosphate dehydrogenase (*GAPDH*) was used for an endogenous control to normalize each RNA samples. All primer sequences are shown in Table 1 [10–15]. 2^-ΔΔCt^ method was used for the relative quantification of target genes.

**Table 1:** Primers used for quantitative real-time PCR

Gene symbol^1^	Primer sequences (from 5′ to 3′)	Length (bp)	Gene Bank ID
OAS1	F: AGAGTCCACGACGGGAGAACC	111	MG799562.1
	R: ACTGACCCAGGGCATCAAAGG		
CD40LG	F: ATTCACTTGGGCGGAGTCTTC	80	HQ110108.1
	R: GTGGCTCACTTGGCTTGGAT		
IL-1B	F: GAAGTGATGGCTAACTACGGTGAC	108	NM_214055.1
	R: TCTCAGAGAACCAAGGTCCAGGT		
IL-6	F: AAAGAATCCAGACAAAGCCACC	83	NM_001252429.1
	R: TCCACTCGTTCTGTGACTGCA		
IL-18R1	F: ATGATTATGTTTTGGAGTTTT	373	NM_214098.1
	R: GTAATATTGAAGGTTTTGGTGA		
GAPDH	F: GCTACACTGAGGACCAGGTTG	294	NM_001206359.1
	R: AGGAGATGCTCGGTGTGTTG		

^1^*OAS1* 2′-5′-Oligoadenylate synthetase 1, *CD40LG* Cluster of differentiation 40 ligand, *IL-1B* Interleukin 1 beta, *IL-6* Interleukin 6, *IL-18R1* Interleukin 18 receptor 1, *GAPDH* Glyceraldehyde 3-phosphate dehydrogenase

### Statistical analysis

All samples for qRT-PCR was run in triplicate and result graph was expressed as means ± S.D. The *P* value was determined using Student’s t test. Pearson’s correlation coefficient was used to measure statistical relationship between qRT-PCR and DNA microarray.

## Results

### Summary of DNA microarray data

A total of 13,673 and 3599 differentially expressed genes (DEGs) were found in each ear skin and placenta (*P* < 0.05, |log_2_ fold| ≥ 1). Of total DEGs, we classified immune-related genes to confirm immunological difference between DPs and GPs. As a result, 90 and 44 genes were found in each ear skin and placenta (Fig. 1b). Specific information was provided in Table S1.

### Comparison of immune related genes in the ear skin and placenta of DP compared to GP

First, we found that the number of up-regulated genes in the ear skin (67/73) was about 7 times greater than that in the placenta (10/73), compared DPs with GPs. However, no significant differences were found in comparison result of down-regulated genes between ear skin (23/53) and placenta (34/53) (Fig. 1c). In addition, we also sorted out 20 immune related genes that were commonly expressed in both tissues for accurate comparison. As a result, the number of up-regulated genes in ear skin (14/20) about 3 times greater than that in placenta (4/20), showing adverse pattern between two tissues (Fig. 1d). Thus, it could be expected that the DPs was infected with pathogens under conventional condition, resulting in immune activation.

### Systemic immune activation in DP

Furthermore, we performed clustering analysis using DNA microarray data of the ear skin to confirm systemic immune activation. Immune related genes were grouped by biological functions that associated with a process of systemic immune activation. Thus, 22 genes were divided into 4 groups (Complement, Innate immunity, T cell signaling, B cell signaling; (Fig. 1e). This result indicated that immune activation was systemically occurred in the body of DPs.

### Validation of DEG data by using quantitative real-time PCR

**Fig. 2:**
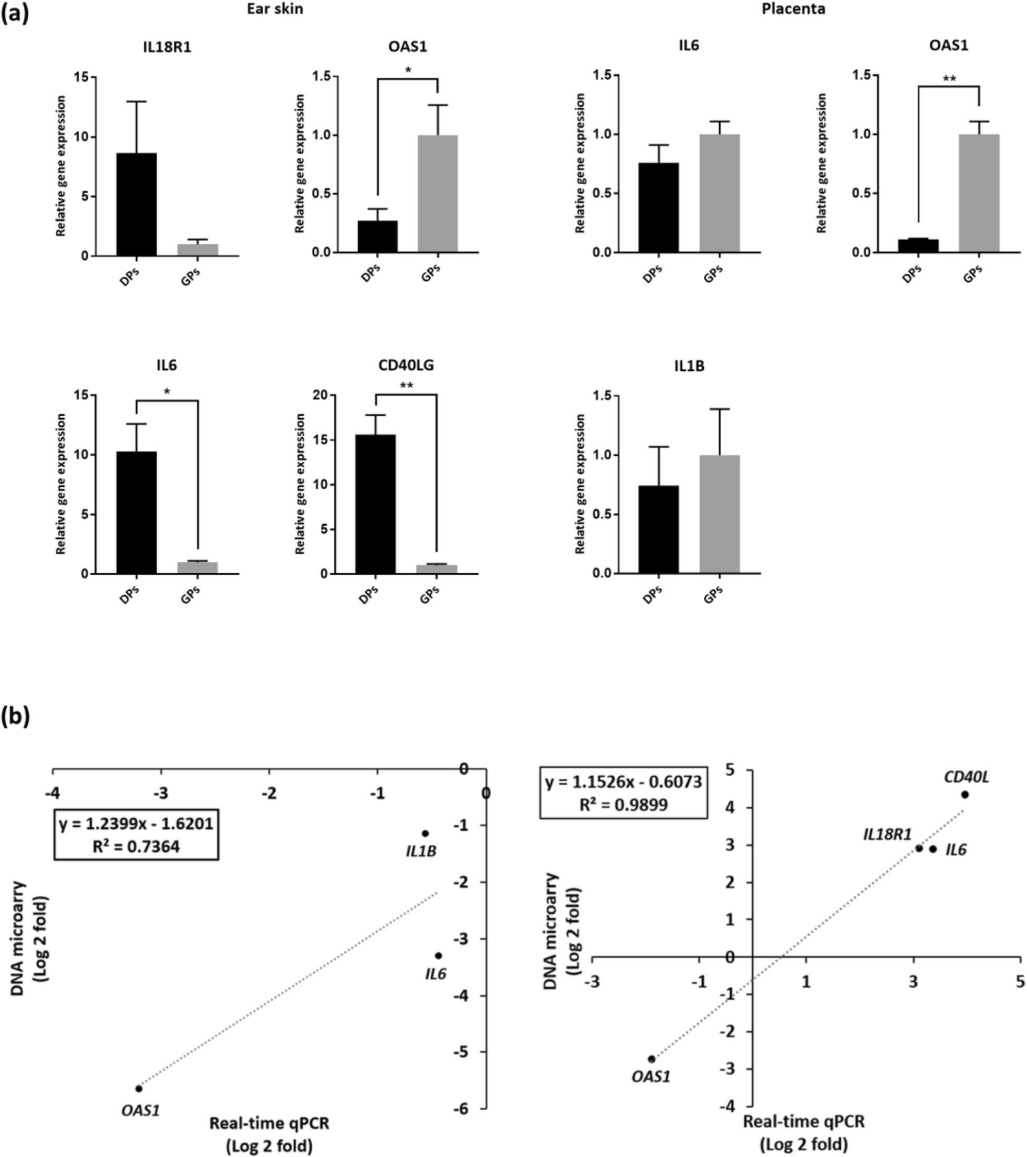
Validation for fold change of DEG in DNA microarray data. **a** qRT-PCR results of candidate genes. Y-axis indicates fold change of gene expression. All samples are run in triplicate. All data are shown as means ± standard deviation. **b** Correlation graph between relative expression of candidate gene from results of qRT-PCR and DNA microarray in the ear skin and the placenta. * *P* ≤ 0.05; ** *P* ≤ 0.01

qRT-PCR was performed with five candidate genes including Interleukin 18 receptor alpha (*IL-18R1*), Cluster of differentiation 40 ligand (*CD40LG*) for ear skin, Interleukin 1 beta (*IL-1B*) for placenta, Interleukin 6 (*IL-6*), and 2′-5′-Oligoadenylate synthetase 1 (*OAS1*) to validate DNA microarray result. As a result, the fold changes of all candidate genes were highly consistent with DNA microarray results (Fig. 2a). Moreover, we calculate correlation coefficient to measure significance of a linear relationship between qRT-PCR and DNA microarray. In conclusion, expression pattern of candidate genes in the ear skin and placenta were highly correlated (Fig. 2b). Specific gene expression result was provided in Table S2.

## Discussion

Our analysis results demonstrated that porcine immune system was affected by microbes in breeding environment. The number of immune-related genes in the ear skin was greater than that in the placenta, which meant that intense immunological changes occur in the marginal tissue (Fig. 1b). We also could verify different expression patterns in placenta, implicating that unidentified mechanism suppress aggressive immune response to prevent its fetus from being killed [16, 17]. According to recent study, it was identified that specific mechanism of immune tolerance between maternal endothelium and fetal tissues during pregnancy [8].

Contrary to expectations, the expression patterns of some genes were not consistent with our conclusion. These results might be explained by the fact that differences of expression value were too small to be statistically significant (Ras-related protein Rab-27A (*RAB27A*), Interleukin 1 beta (*IL-1B*), KIT proto-oncogene, receptor tyrosine kinase (*KIT*), and MHC class II histocompatibility antigen SLA-DQA (*SLA-DQA1*)). In addition, homologous expression pattern of Colony stimulating factor 2 (*CSF2*), Vitronectin (*VTN*), and *OAS1* in both ear skin and placenta or increased expression of immune genes in the placenta of germ-free minipigs have not been clearly explained. However, some studies reported that expression of some immune genes in placenta could be unexpected because of variable reasons, such as epigenetic modification and its unique microenvironment [18, 19]. In addition, pregnant mice raised in conventional and germ-free environment represented different immunological adaptations [20]. These studies suggested that un identified immune modulation were occurred during pregnancy.

## Conclusions

In spite of some experimental limitations, we identified that uncontrolled breeding facility could spoil immune system of experimental animals. Clinically, porcine skin have been well known for good model in toxicology studies due to its similarity of histological structure and skin immune system with human [21]. This study could support the idea that researchers should breed minipigs in the bacteria-controlled facility to perform qualified animal experiment.

## Supplementary Information

**Additional file 1: Supplementary table 1.** Summary of DEGs from DNA microarray data.

**Additional file 2: Supplementary table 2.** Gene information of systemic immune activation.

## Data Availability

All experiment data during this study are included in this manuscript and supporting files.

## Abbreviations

DP: Domestic pig
GP: Germ free minipig
GO: Gene ontology
GLP: Good laboratory practice
qRT-PCR: Quantitative real-time PCR
DEG: Differentially expressed gene
IL-18R1: Interleukin 18 receptor alpha
CD40LG: Cluster of differentiation 40 ligand
IL-6: Interleukin 6
OAS1: 2′-5-Oligoadenylate synthetase 1
RAB27A: Ras-related protein Rab-27A
KIT: KIT proto-oncogene, receptor tyrosine kinase
SLA-DQA1: MHC class II histocompatibility antigen SLA-DQA
CSF2: Colony stimulating factor 2
VTN: Vitronectin
IL-1B: Interleukin 1 beta
