# Supplementary material for: Comparative analysis of immune related genes between domestic pig and germ-free minipig
Source: Lab Anim Res. 2020 Dec 1;36:44. doi: 10.1186/s42826-020-00077-7 (PMC7709342; doi:10.1186/s42826-020-00077-7)
Supplement: Supplementary file 1 — Additional file 1: Supplementary table 1. Summary of DEGs from DNA microarray data. [file 42826_2020_77_MOESM1_ESM.docx]

**Supplementary table 1.** Summary of DEGs from DNA microarray data

| **Tissue** | **Total DEG**^1^ | | |  | **Immune-related genes** | | |
| --- | --- | --- | --- | --- | --- | --- | --- |
|  | **> 2 fold** | **> 4 fold** | **> 8 fold** |  | **> 2 fold** | **> 4 fold** | **> 8 fold** |
| Ear skin | 13673 | 8623 | 4712 |  | 90 | 49 | 28 |
| Placenta | 3599 | 727 | 188 |  | 44 | 11 | 4 |

^1^Selection criteria for DEG is more than 2 fold change of gene expression.
