# Supplementary material for: Comparative analysis of immune related genes between domestic pig and germ-free minipig
Source: Lab Anim Res. 2020 Dec 1;36:44. doi: 10.1186/s42826-020-00077-7 (PMC7709342; doi:10.1186/s42826-020-00077-7)
Supplement: Supplementary file 2 — Additional file 2: Supplementary table 2. Gene information of systemic immune activation. [file 42826_2020_77_MOESM2_ESM.docx]

**Supplementary table 2.** Gene information of systemic immune activation.

| **Gene symbol** | **Gene name** | **Accession no.** | **Log_2_ fold change^1^** | **Biological function** |
| --- | --- | --- | --- | --- |
| C5 | Complement C5 | NM_001001646 | 2.47 | C3 complement |
| C6 | Complement C6 | NM_001097449 | 2.62 |  |
| C7 | Complement C7 | NM_214282 | 2.05 |  |
| C8A | Complement C8 alpha chain | NM_001097450 | 4.66 |  |
| MASP1 | Mannan binding lectin serine peptidase 1 | NM_001184947 | 4.02 |  |
| MBL1 | Mannose binding lectin (A) | NM_001007194 | 4.81 |  |
| MBL2 | Mannose binding lectin 2 | NM_214125 | 2.83 |  |
| IL6 | Interleukin 6 | NM_214399 | 2.89 | Innate immunity |
| IL23A | Interleukin 23 subunit alpha | NM_001130236 | 4.67 |  |
| ISG20 | Interferon stimulated exonuclease gene 20 | NM_001005351 | 4.13 |  |
| SYK | Spleen associated tyrosine kinase | NM_001104952 | 3.12 |  |
| ULBP1 | UL16 binding protein 1 | NM_001004035 | 2.31 |  |
| CD4 | CD4 molecule | NM_001001908 | 4.23 | T cell signaling |
| CD40LG | CD40 ligand | NM_214126 | 4.35 |  |
| CSF2 | Colony stimulating factor 2 | NM_214118 | 3.18 |  |
| CTLA4 | Cytotoxic T-lymphocyte associated protein 4 | NM_214149 | 3.89 |  |
| IL4 | Interleukin 4 | NM_214123 | 3.96 |  |
| CD19 | CD19 molecule | NM_214377 | 3.98 | B cell signaling |
| CD1D | CD1d molecule | NM_001102680 | 3.14 |  |
| IL7R | Interleukin 7 receptor | NM_001146128 | 2.17 |  |
| IL12RB1 | Interleukin 12 receptor subunit beta 1 | NM_001145986 | 3.44 |  |
| IL18RA | Interleukin 18 receptor 1 | NM_214098 | 3.13 |  |
| NFATC2 | Nuclear factor of activated T cells 2 | NM_001113452 | 2.21 |  |
| SLA-DOB | MHC class II, DO beta | NM_001114064 | 3.45 |  |

^1^Fold change derived from microarray data of ear skin, compared domestic pig to germ-free minipig.
